# *catena*-Poly[[[tetra­aqua­zinc(II)]-μ-1,4-bis­(1,2,4-triazol-1-yl)butane-κ^2^ *N* ^4^:*N* ^4′^] biphenyl-4,4′-dicarboxyl­ate]

**DOI:** 10.1107/S160053681003970X

**Published:** 2010-10-13

**Authors:** Chang-Mei Jiao

**Affiliations:** a Department of Chemistry, Yancheng Teachers’ College, Yancheng 224002, People’s Republic of China

## Abstract

The asymmetric unit of the polymeric title compound, {[Zn(C_8_H_12_N_6_)(H_2_O)_4_](C_14_H_8_O_4_)}_*n*_ or {[Zn(BTB)(H_2_O)_4_](BPDC)}_*n*_ [BTB is 1,4-bis­(1,2,4-triazol-1-yl)butane and H_2_BPDC is biphenyl-4,4′-dicarb­oxy­lic acid], contains half a [Zn(BTB)(H_2_O)_4_]^2+^ cation and half a BPDC anion, both ions lying about a crystallographic inversion centre. The crystal structure consists of zigzag polymeric cationic chains parallel to the *c* axis and uncoordinated anions linked into a three-dimensional supra­molecular architecture by O—H⋯O, C—H⋯O hydrogen bonds and C—H⋯π inter­actions.

## Related literature

For general background to the structures and applications of supra­molecular compounds, see: Kitagawa *et al.* (2004 ▶); Ferey *et al.* (2005 ▶); Roy *et al.* (2009 ▶); Zhang *et al.* (2009 ▶). For related compounds based on 1,4-bis­(1,2,4-triazol-1-yl)butane, see: Liu *et al.* (2008 ▶); Gu *et al.* (2008 ▶); Wang *et al.* (2008 ▶); Zhu *et al.* (2009 ▶). 
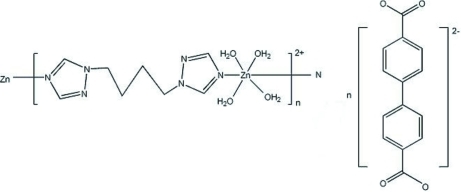


## Experimental

### 

#### Crystal data

[Zn(C_8_H_12_N_6_)(H_2_O)_4_](C_14_H_8_O_4_)
*M* *_r_* = 569.89
Triclinic, 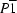

*a* = 6.4344 (15) Å
*b* = 7.1490 (18) Å
*c* = 13.539 (3) Å
α = 89.250 (4)°
β = 81.348 (4)°
γ = 72.620 (3)°
*V* = 587.3 (2) Å^3^
*Z* = 1
Mo *K*α radiation
μ = 1.11 mm^−1^
*T* = 293 K
0.21 × 0.19 × 0.17 mm

#### Data collection

Bruker SMART APEX CCD diffractometer
Absorption correction: multi-scan (*SADABS*; Bruker, 2000 ▶) *T*_min_ = 0.792, *T*_max_ = 0.828
3162 measured reflections
2299 independent reflections
1788 reflections with *I* > 2σ(*I*)
*R*_int_ = 0.032

#### Refinement

*R*[*F* ^2^ > 2σ(*F* ^2^)] = 0.040
*wR*(*F* ^2^) = 0.080
*S* = 0.92
2237 reflections
169 parameters
H-atom parameters constrained
Δρ_max_ = 0.47 e Å^−3^
Δρ_min_ = −0.31 e Å^−3^

### 

Data collection: *SMART* (Bruker, 2000 ▶); cell refinement: *SAINT* (Bruker, 2000 ▶); data reduction: *SAINT*; program(s) used to solve structure: *SHELXS97* (Sheldrick, 2008 ▶); program(s) used to refine structure: *SHELXL97* (Sheldrick, 2008 ▶); molecular graphics: *DIAMOND* (Brandenburg, 1999 ▶); software used to prepare material for publication: *SHELXTL* (Sheldrick, 2008 ▶).

## Supplementary Material

Crystal structure: contains datablocks I, global. DOI: 10.1107/S160053681003970X/rz2494sup1.cif

Structure factors: contains datablocks I. DOI: 10.1107/S160053681003970X/rz2494Isup2.hkl

Additional supplementary materials:  crystallographic information; 3D view; checkCIF report

## Figures and Tables

**Table 1:** Hydrogen-bond geometry (Å, °) *Cg* is the centroid of the C6–C11 benzene ring.

*D*—H⋯*A*	*D*—H	H⋯*A*	*D*⋯*A*	*D*—H⋯*A*
C2—H2*A*⋯O4^i^	0.93	2.50	3.342 (3)	150
O1—H1*D*⋯O3^ii^	0.85	2.07	2.825 (3)	148
O1—H1*C*⋯O3	0.85	1.95	2.783 (2)	167
O2—H2*C*⋯O4^iii^	0.85	1.85	2.642 (3)	155
O2—H2*D*⋯O3	0.85	2.06	2.839 (3)	151
C3—H3*B*⋯*Cg*	0.97	2.82	3.552 (3)	133

Symmetry codes: (i) 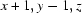; (ii) 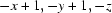; (iii) 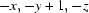.

**Fig. 1.**
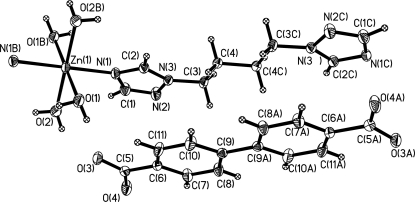
A view of the structure of the title compound, showing the atom-numbering scheme and the coordination geometry around the zinc(II) centre. Displacement ellipsoids are drawn at the 50% probability level and H atoms are shown as small spheres of arbitrary radii. [Symmetry codes: (A) 1-x, 1-y, 1-z; (B) 1-x, -y, -z; (C) 1-x, -y, 1-z]

**Fig. 2.**
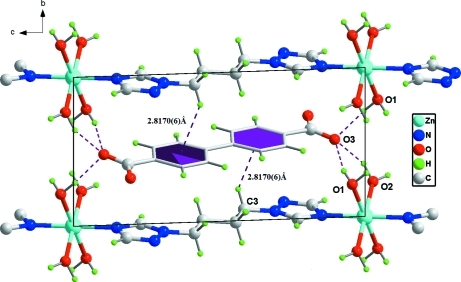
A perspective view of the two-dimensional supramolecular sheet of the title compound along the a axis, showing intermolecular O—H···O hydrogen bonds and C—H···π interactions (dashed lines).

**Fig. 3.**
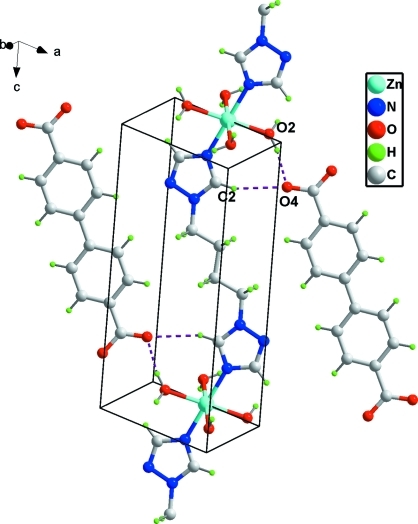
The three-dimensional network of the title compound. Dashed lines indicate hydrogen bonds.

## supplementary crystallographic information

### Comment

Interest in crystal engineering and supramolecular chemistry is rapidly increasing not only because of their fascinating structures and topologies but also owing to their potential use in optical, electrical, catalytic and adsorptive applications (Kitagawa *et al.*, 2004; Ferey *et al.*, 2005; Roy *et al.*, 2009; Zhang *et al.*, 2009). The construction of such coordination polymers is highly influenced by several factors. However, the key factor in the manipulating coordination polymers is undoubtedly the selection of appropriate ligands. Comparing to rigid ligands, bifunctional flexible ligands can induce variability of the structure and may lead to the formation of supramolecular isomers because of their conformational flexibility. Recently, a series of transition metal coordination polymers based on the flexible ligand 1,4-bis(1,2,4-triazol-1-yl)butane have been reported (Liu *et al.*, 2008; Gu *et al.*, 2008; Wang *et al.*, 2008; Zhu *et al.*, 2009). In this paper, biphenyl-4,4'-dicarboxylic acid (H_2_BPDC) and 1,4-bis(1,2,4-triazol-1-yl)butane (BTB) have been selected as organic linkers, generating the title new zinc(II) coordination polymer, (I), the crystal structure of which is reported herein.

Compound (I) crystallizes in the triclinic space group P -1, and the asymmetric unit contains half a [Zn(C_8_H_12_N_6_)(H_2_O)_4_]^2+^ cation and half a uncoordinated BPDC anion. Each zinc(II) metal is located on an inversion centre and is six-coordinated in a octahedron geometry by two triazole nitrogen atoms from two different BTB ligand in the axial positions, and four oxygen atom from coordinated water molecules at the equatorial plane (Figure 1). The Zn–N bond length is 2.096 (2) Å, while the Zn–O bond lengths are 2.1234 (18) Å, 2.1693 (19)Å respectively. The BTB ligand adopts a *trans–trans-trans* conformation and acts as a *N*,*N*'-bidentate ligand linking centrosymmetrically-related zinc(II) cations into one-dimensional *zig-zag* cationic chains parallel to the *c* axis. The doubly deprotonated BPDC anion, which has crystallographically imposed centre of symmetry, does not coordinate to the zinc(II) centres and only acts as counter-ion. The anionic and cationic parts of (I) interact to form a three-dimensional network through intermolecular interactions such as conventional O—H···O hydrogen bonds, non-conventional C—H···O contacts and C—H···π interactions (Table 1). The C—H···π interaction is observed between the H3B atom and the centroid of the C6–C11 ring. As shown in Figure 2, interchain O1—H1C···O3, O1—H1D···O3, O2—H2D···O3 bonds between coordinated water molecules and the carboxylate anion are found to assemble the 1-D motifs into a 2-D layer parallel to the *bc* plane. These layers are further connected by interlayer O2—H2C···O4, C2—H2A···O4 hydrogen bonds, forming a 3-D supramolecular network (Figure 3).

### Experimental

A mixture of Zn(NO_3_)_2_.6H_2_O (29.7 mg, 0.1 mmol), biphenyl-4,4'-dicarboxylic acid (H_2_BPDC) (24.2 mg, 0.1 mmol), 1,4-bis(1,2,4-triazol-1-yl)butane (BTB) (19.2 mg, 0.1 mmol), and KOH (11.2 mg, 0.2 mmol) in H_2_O (10 ml) was sealed in a 16 ml Teflon-lined stainless steel container and heated at 180°C for 72 h. After cooling to room temperature, white block crystals of the title compound were collected by filtration and washed with water and ethanol several times (yield 51.3%, based on H_2_BPDC). Elemental analysis for C_22_H_28_ZnN_6_O_8_ (Mr = 569.87): C 46.37, H 4.95, N 14.75%; found: 46.46, H 4.98, N 14.79%.

### Refinement

The water H atoms were located in a difference Fourier map and fixed in the refinement, with *U*_iso_(H)=1.2*U*_eq_(O). All C-bound H atoms were placed in calculated positions and refined using a riding model, with C—H = 0.93 (triazole, aromatic) or 0.97 Å(methylene) and *U_iso_*(H) = 1.2*U_eq_*(C).

### Crystal data

**Table:**

[Zn(C_8_H_12_N_6_)(H_2_O)_4_](C_14_H_8_O_4_)	*Z* = 1
*M**_r_* = 569.89	*F*(000) = 296
Triclinic, *P*1	*D*_x_ = 1.611 Mg m^−^^3^
Hall symbol: -P 1	Mo *K*α radiation, λ = 0.71073 Å
*a* = 6.4344 (15) Å	Cell parameters from 1152 reflections
*b* = 7.1490 (18) Å	θ = 3.0–25.0°
*c* = 13.539 (3) Å	µ = 1.11 mm^−^^1^
α = 89.250 (4)°	*T* = 293 K
β = 81.348 (4)°	Block, white
γ = 72.620 (3)°	0.21 × 0.19 × 0.17 mm
*V* = 587.3 (2) Å^3^	

### Data collection

**Table:**

Bruker SMART APEX CCD diffractometer	2299 independent reflections
Radiation source: fine-focus sealed tube	1788 reflections with *I* > 2σ(*I*)
graphite	*R*_int_ = 0.032
phi and ω scans	θ_max_ = 26.0°, θ_min_ = 3.0°
Absorption correction: multi-scan (*SADABS*; Bruker, 2000)	*h* = −6→7
*T*_min_ = 0.792, *T*_max_ = 0.828	*k* = −6→8
3162 measured reflections	*l* = −16→16

### Refinement

**Table:**

Refinement on *F*^2^	Primary atom site location: structure-invariant direct methods
Least-squares matrix: full	Secondary atom site location: difference Fourier map
*R*[*F*^2^ > 2σ(*F*^2^)] = 0.040	Hydrogen site location: inferred from neighbouring sites
*wR*(*F*^2^) = 0.080	H-atom parameters constrained
*S* = 0.92	*w* = 1/[σ^2^(*F*_o_^2^) + (0.0317*P*)^2^] where *P* = (*F*_o_^2^ + 2*F*_c_^2^)/3
2237 reflections	(Δ/σ)_max_ < 0.001
169 parameters	Δρ_max_ = 0.47 e Å^−^^3^
0 restraints	Δρ_min_ = −0.31 e Å^−^^3^

### Special details

**Table:**

Geometry. All e.s.d.'s (except the e.s.d. in the dihedral angle between two l.s. planes) are estimated using the full covariance matrix. The cell e.s.d.'s are taken into account individually in the estimation of e.s.d.'s in distances, angles and torsion angles; correlations between e.s.d.'s in cell parameters are only used when they are defined by crystal symmetry. An approximate (isotropic) treatment of cell e.s.d.'s is used for estimating e.s.d.'s involving l.s. planes.
Refinement. Refinement of *F*^2^ against ALL reflections. The weighted *R*-factor *wR* and goodness of fit *S* are based on *F*^2^, conventional *R*-factors *R* are based on *F*, with *F* set to zero for negative *F*^2^. The threshold expression of *F*^2^ > σ(*F*^2^) is used only for calculating *R*-factors(gt) *etc*. and is not relevant to the choice of reflections for refinement. *R*-factors based on *F*^2^ are statistically about twice as large as those based on *F*, and *R*- factors based on ALL data will be even larger.

### Fractional atomic coordinates and isotropic or equivalent isotropic displacement parameters (Å^2^)

**Table:**

	*x*	*y*	*z*	*U*_iso_*/*U*_eq_	
C1	0.1236 (5)	0.0898 (4)	0.1819 (2)	0.0408 (7)	
H1A	0.0180	0.1181	0.1395	0.049*	
C2	0.4301 (4)	−0.0006 (4)	0.23078 (18)	0.0323 (6)	
H2A	0.5802	−0.0473	0.2337	0.039*	
C3	0.2822 (4)	0.0747 (4)	0.41485 (17)	0.0347 (7)	
H3A	0.1835	0.0101	0.4519	0.042*	
H3B	0.2282	0.2125	0.4350	0.042*	
C4	0.5070 (4)	−0.0100 (4)	0.44352 (17)	0.0311 (6)	
H4A	0.6059	0.0584	0.4103	0.037*	
H4B	0.5653	−0.1473	0.4224	0.037*	
C5	0.1402 (5)	0.5973 (4)	0.18630 (19)	0.0319 (6)	
C6	0.2502 (4)	0.5703 (4)	0.27840 (17)	0.0275 (6)	
C7	0.1272 (4)	0.6255 (4)	0.37179 (19)	0.0375 (7)	
H7	−0.0245	0.6825	0.3773	0.045*	
C8	0.2241 (4)	0.5981 (4)	0.45708 (19)	0.0367 (7)	
H8	0.1358	0.6366	0.5187	0.044*	
C9	0.4489 (4)	0.5149 (3)	0.45388 (17)	0.0262 (6)	
C10	0.5721 (4)	0.4649 (4)	0.35941 (19)	0.0389 (7)	
H10	0.7243	0.4120	0.3534	0.047*	
C11	0.4747 (5)	0.4915 (4)	0.27460 (19)	0.0393 (7)	
H11	0.5629	0.4552	0.2128	0.047*	
N1	0.3416 (3)	0.0186 (3)	0.14818 (15)	0.0333 (6)	
N2	0.0731 (4)	0.1156 (3)	0.27853 (16)	0.0416 (6)	
N3	0.2730 (4)	0.0566 (3)	0.30894 (15)	0.0299 (5)	
O1	0.6429 (3)	0.2192 (3)	0.04395 (12)	0.0405 (5)	
H1C	0.5383	0.3192	0.0681	0.049*	
H1D	0.7103	0.2541	−0.0085	0.049*	
O2	0.2224 (3)	0.2334 (3)	−0.02699 (13)	0.0459 (5)	
H2C	0.2093	0.2385	−0.0886	0.055*	
H2D	0.2284	0.3433	−0.0065	0.055*	
O3	0.2593 (3)	0.5246 (3)	0.10408 (13)	0.0407 (5)	
O4	−0.0583 (3)	0.6896 (3)	0.19568 (13)	0.0484 (6)	
Zn1	0.5000	0.0000	0.0000	0.03341 (17)	

### Atomic displacement parameters (Å^2^)

**Table:**

	*U*^11^	*U*^22^	*U*^33^	*U*^12^	*U*^13^	*U*^23^
C1	0.0328 (18)	0.0533 (19)	0.0323 (16)	−0.0054 (14)	−0.0083 (13)	0.0027 (13)
C2	0.0283 (15)	0.0366 (16)	0.0266 (14)	−0.0028 (13)	−0.0014 (12)	0.0002 (12)
C3	0.0380 (17)	0.0397 (16)	0.0211 (14)	−0.0057 (13)	0.0000 (12)	−0.0039 (12)
C4	0.0349 (16)	0.0298 (15)	0.0243 (14)	−0.0051 (12)	−0.0007 (12)	−0.0001 (11)
C5	0.0369 (18)	0.0294 (15)	0.0293 (15)	−0.0086 (13)	−0.0078 (13)	0.0016 (11)
C6	0.0302 (15)	0.0275 (14)	0.0246 (13)	−0.0069 (12)	−0.0072 (11)	0.0009 (11)
C7	0.0236 (15)	0.0506 (18)	0.0302 (15)	0.0012 (13)	−0.0049 (12)	0.0033 (13)
C8	0.0284 (16)	0.0504 (18)	0.0238 (14)	−0.0026 (13)	0.0000 (12)	0.0025 (12)
C9	0.0246 (15)	0.0250 (14)	0.0285 (14)	−0.0058 (11)	−0.0057 (11)	0.0005 (11)
C10	0.0222 (15)	0.0561 (19)	0.0311 (15)	−0.0005 (13)	−0.0045 (12)	−0.0085 (13)
C11	0.0314 (17)	0.0554 (19)	0.0238 (14)	−0.0041 (14)	0.0008 (12)	−0.0086 (12)
N1	0.0317 (14)	0.0398 (14)	0.0238 (12)	−0.0028 (11)	−0.0058 (10)	0.0009 (10)
N2	0.0296 (14)	0.0589 (16)	0.0300 (13)	−0.0044 (12)	−0.0035 (11)	0.0003 (11)
N3	0.0286 (13)	0.0324 (13)	0.0247 (11)	−0.0038 (10)	−0.0027 (10)	−0.0013 (9)
O1	0.0406 (12)	0.0466 (12)	0.0289 (10)	−0.0073 (10)	0.0000 (9)	−0.0030 (9)
O2	0.0524 (13)	0.0422 (12)	0.0344 (11)	0.0043 (10)	−0.0178 (9)	−0.0028 (9)
O3	0.0408 (12)	0.0480 (12)	0.0257 (10)	−0.0010 (10)	−0.0059 (9)	−0.0036 (9)
O4	0.0322 (12)	0.0707 (15)	0.0306 (11)	0.0062 (11)	−0.0125 (9)	−0.0015 (10)
Zn1	0.0329 (3)	0.0407 (3)	0.0206 (2)	−0.0017 (2)	−0.00443 (19)	−0.00010 (19)

### Geometric parameters (Å, °)

**Table:**

C1—N2	1.301 (3)	C7—H7	0.9300
C1—N1	1.350 (3)	C8—C9	1.384 (4)
C1—H1A	0.9300	C8—H8	0.9300
C2—N1	1.317 (3)	C9—C10	1.389 (3)
C2—N3	1.323 (3)	C9—C9^ii^	1.481 (5)
C2—H2A	0.9300	C10—C11	1.374 (4)
C3—N3	1.453 (3)	C10—H10	0.9300
C3—C4	1.500 (4)	C11—H11	0.9300
C3—H3A	0.9700	N1—Zn1	2.096 (2)
C3—H3B	0.9700	N2—N3	1.354 (3)
C4—C4^i^	1.524 (5)	O1—Zn1	2.1693 (19)
C4—H4A	0.9700	O1—H1C	0.8500
C4—H4B	0.9700	O1—H1D	0.8499
C5—O4	1.238 (3)	O2—Zn1	2.1234 (18)
C5—O3	1.272 (3)	O2—H2C	0.8500
C5—C6	1.507 (4)	O2—H2D	0.8500
C6—C11	1.378 (4)	Zn1—N1^iii^	2.096 (2)
C6—C7	1.381 (3)	Zn1—O2^iii^	2.1234 (18)
C7—C8	1.378 (4)	Zn1—O1^iii^	2.1693 (19)
			
N2—C1—N1	114.6 (3)	C11—C10—H10	119.2
N2—C1—H1A	122.7	C9—C10—H10	119.2
N1—C1—H1A	122.7	C10—C11—C6	122.0 (2)
N1—C2—N3	109.7 (2)	C10—C11—H11	119.0
N1—C2—H2A	125.1	C6—C11—H11	119.0
N3—C2—H2A	125.1	C2—N1—C1	103.1 (2)
N3—C3—C4	114.8 (2)	C2—N1—Zn1	128.25 (18)
N3—C3—H3A	108.6	C1—N1—Zn1	127.55 (18)
C4—C3—H3A	108.6	C1—N2—N3	102.5 (2)
N3—C3—H3B	108.6	C2—N3—N2	110.0 (2)
C4—C3—H3B	108.6	C2—N3—C3	131.7 (2)
H3A—C3—H3B	107.5	N2—N3—C3	118.2 (2)
C3—C4—C4^i^	109.7 (3)	Zn1—O1—H1C	108.1
C3—C4—H4A	109.7	Zn1—O1—H1D	107.9
C4^i^—C4—H4A	109.7	H1C—O1—H1D	107.4
C3—C4—H4B	109.7	Zn1—O2—H2C	111.6
C4^i^—C4—H4B	109.7	Zn1—O2—H2D	111.7
H4A—C4—H4B	108.2	H2C—O2—H2D	109.6
O4—C5—O3	124.7 (2)	N1^iii^—Zn1—N1	180.00 (5)
O4—C5—C6	118.0 (2)	N1^iii^—Zn1—O2	93.74 (8)
O3—C5—C6	117.3 (2)	N1—Zn1—O2	86.26 (8)
C11—C6—C7	116.7 (2)	N1^iii^—Zn1—O2^iii^	86.26 (8)
C11—C6—C5	122.7 (2)	N1—Zn1—O2^iii^	93.74 (8)
C7—C6—C5	120.6 (2)	O2—Zn1—O2^iii^	180.00 (9)
C8—C7—C6	121.5 (2)	N1^iii^—Zn1—O1	93.19 (8)
C8—C7—H7	119.3	N1—Zn1—O1	86.81 (8)
C6—C7—H7	119.3	O2—Zn1—O1	87.85 (8)
C7—C8—C9	122.0 (2)	O2^iii^—Zn1—O1	92.15 (8)
C7—C8—H8	119.0	N1^iii^—Zn1—O1^iii^	86.81 (8)
C9—C8—H8	119.0	N1—Zn1—O1^iii^	93.19 (8)
C8—C9—C10	116.1 (2)	O2—Zn1—O1^iii^	92.15 (8)
C8—C9—C9^ii^	121.5 (3)	O2^iii^—Zn1—O1^iii^	87.85 (8)
C10—C9—C9^ii^	122.4 (3)	O1—Zn1—O1^iii^	180.00 (10)
C11—C10—C9	121.7 (2)		
			
N3—C3—C4—C4^i^	−177.5 (3)	N2—C1—N1—C2	0.1 (3)
O4—C5—C6—C11	−171.9 (3)	N2—C1—N1—Zn1	−168.61 (19)
O3—C5—C6—C11	7.6 (4)	N1—C1—N2—N3	0.0 (3)
O4—C5—C6—C7	7.9 (4)	N1—C2—N3—N2	0.2 (3)
O3—C5—C6—C7	−172.7 (2)	N1—C2—N3—C3	−176.2 (2)
C11—C6—C7—C8	−1.7 (4)	C1—N2—N3—C2	−0.1 (3)
C5—C6—C7—C8	178.6 (2)	C1—N2—N3—C3	176.9 (2)
C6—C7—C8—C9	0.3 (4)	C4—C3—N3—C2	−9.0 (4)
C7—C8—C9—C10	1.5 (4)	C4—C3—N3—N2	174.8 (2)
C7—C8—C9—C9^ii^	−179.6 (3)	C2—N1—Zn1—O2	−141.6 (2)
C8—C9—C10—C11	−1.8 (4)	C1—N1—Zn1—O2	24.4 (2)
C9^ii^—C9—C10—C11	179.3 (3)	C2—N1—Zn1—O2^iii^	38.4 (2)
C9—C10—C11—C6	0.4 (5)	C1—N1—Zn1—O2^iii^	−155.6 (2)
C7—C6—C11—C10	1.3 (4)	C2—N1—Zn1—O1	−53.5 (2)
C5—C6—C11—C10	−178.9 (3)	C1—N1—Zn1—O1	112.5 (2)
N3—C2—N1—C1	−0.2 (3)	C2—N1—Zn1—O1^iii^	126.5 (2)
N3—C2—N1—Zn1	168.47 (17)	C1—N1—Zn1—O1^iii^	−67.5 (2)

Symmetry codes: (i) −*x*+1, −*y*, −*z*+1; (ii) −*x*+1, −*y*+1, −*z*+1; (iii) −*x*+1, −*y*, −*z*.

### Hydrogen-bond geometry (Å, °)

**Table:**

*Cg* is the centroid of the C6–C11 benzene ring.

**Table:**

*D*—H···*A*	*D*—H	H···*A*	*D*···*A*	*D*—H···*A*
C2—H2A···O4^iv^	0.93	2.50	3.342 (3)	150
O1—H1D···O3^v^	0.85	2.07	2.825 (3)	148
O1—H1C···O3	0.85	1.95	2.783 (2)	167
O2—H2C···O4^vi^	0.85	1.85	2.642 (3)	155
O2—H2D···O3	0.85	2.06	2.839 (3)	151
C3—H3B···Cg	0.97	2.82	3.552 (3)	133

Symmetry codes: (iv) *x*+1, *y*−1, *z*; (v) −*x*+1, −*y*+1, −*z*; (vi) −*x*, −*y*+1, −*z*.
